# Letter to editor: confusions about the role of RNF149 in apoptosis and PI3K/AKT signaling

**DOI:** 10.1007/s12032-023-02213-4

**Published:** 2023-10-20

**Authors:** Xiaobin Guo, Qian Yang, Hongxin Yang

**Affiliations:** Department of pharmacy, Inner Mongolia Autonomous Region People’s Hospital, Hohhot, Inner Mongolia 010017 PR China

Dear Editor,

We read with great interest the article of Zhu et al. [1]titled “RNF149 confers cisplatin resistance in esophageal squamous cell carcinoma via destabilization of PHLPP2 and activating PI3K/AKT signaling " in a recent issue of the journal. In this study, a lot of work has been done on the function and the mechanism of RNF149 in ESCC. However, we have some queries on the article.

Zhu et al. proposed RNF149 overexpression may contribute to apoptosis resistance role in ESCC via activating AKT signaling pathway. The results of Real-time PCR they demonstrated in Fig. 4D are confusing. It is well known that BIM is a pro-apoptotic member of the BCL−2 family, and BCL−2 is an anti-apoptotic member of the same family [2]. While in Zhu’s study, RNA expression levels of BIM and BCL−2 all increased in RNF149 overexpression ESCC cells, and all decreased in RNF149 downregulation cells. XIAP has the anti-apoptotic activity with negatively regulating the activation of caspase 3 [2]. But in Zhu’s study, XIAP deceased in RNF149 overexpression ESCC cells, and increased by downregulation of RNF149. Based on the conclusion of RNF149 producing apoptosis resistance, the results about RNA expression of BIM and XIAP are hard to understand. p27 and p21 are thought to suppress tumor growth and prevent cell cycle progression [3]. However, in Zhu’s study, RNA expression levels of p27 and p21 increased in RNF149 overexpression ESCC cells, and all decreased in RNF149 downregulation cells. The results do not support the conclusion “overexpression or inhibition of RNF149 confers CDDP resistance or sensitivity to ESCC”. In this part, the author proved the influence of RNF149 on expression of AKT downstream genes, but ignored relationship between these genes and apoptosis and cell cycle. This results in confusions about the role of RNF149 in apoptosis and cell cycle and PI3K/AKT signaling.

Another query is, in Fig. 2B, stably expressed RNF149 and silencing RNF149 caused an increase or decrease in Eca109 cells respectively, while RNF149 protein expression level decreased or increased unexpectedly in RNF149 overexpression or downregulation Kyse510 cells. This may be an error.

Yours Sincerely.
